# Clues cue the smooze: rhyme, pausing, and prediction help children learn new words from storybooks

**DOI:** 10.3389/fpsyg.2014.00149

**Published:** 2014-02-21

**Authors:** Kirsten Read

**Affiliations:** Department of Psychology, Santa Clara UniversitySanta Clara, CA, USA

**Keywords:** shared storybook reading, word learning, vocabulary growth, rhyme, predictable language, preschoolers

## Abstract

Rhyme, which is ubiquitous in the language experiences of young children, may be especially facilitative to vocabulary learning because of how it can support active predictions about upcoming words. In two experiments, we tested whether rhyme, when used to help children anticipate new words would make those words easier to learn. Two- to 4-year-old children heard rhyming stanzas naming novel monsters under three conditions: A non-rhyme condition in which novel monster names appeared as unrhymed elements within a rhymed stanza, a non-predictive rhyme condition in which the novel names were the rhymed element in the first line of a stanza, and a predictive rhyme condition in which the monster name came as the rhymed element in the last line of the stanza after a description of the features that distinguished him. In tests of retention and identification children showed greatest novel name learning in the predictive rhyme condition in both between-subjects (Experiment 1) and within-subjects (Experiment 2) comparisons. Additionally, when parents acted as the storybook readers in Experiment 2, many of them distinctly paused before target words in the predictive rhyme condition and for their children a stronger predictive rhyme advantage surfaced. Thus rhyme is not only facilitative for learning, but when the novel vocabulary is specifically in a position where it is *predictable* from the rhymes, it is most accessible.

## Introduction

Recently, my 3-year-old son asked, “Guess what's in my pocket?” then paused for a beat and exclaimed, “a wocket!” And, while thankfully there was not actually a small Seussian monster in his pants, my son had reminded me of just how well he is able to learn words (even nonsense words) from storybooks, and in fact just how strongly rhyme must influence that process.

Shared storybook reading is a triangular interaction—the adult reader, the child, and the book conspire to create a meaningful experience each time a book is shared; and one of the many benefits for young children who regularly share in storybooks with their parents is the positive impact that has on their vocabulary growth (Senechal et al., 1996; DeTemple and Snow, 2003; Blewitt et al., 2009; Farrant and Zubrick, 2013). Books present new words to young listeners, parents actively highlight those new words, expand on them, and help to make them memorable for the child (e.g., Clark, 2010). Even in the earliest years caregivers use storybooks as a vehicle for pointing out new vocabulary (Ninio, 1983; Moerk, 1985; Fletcher and Reese, 2005), but for 2- to 5-year-olds in a rapid period of word learning, storybook reading is the perfect opportunity to hear an abundance of new words in engaging contexts, and to take part in questions and conversations which promote vocabulary learning (Ard and Beverly, 2004; Blewitt et al., 2009).

Of course, the connection between storybook reading and vocabulary growth is not always a simple one. Learning words from books is mediated by many factors—the frequency of reading (Bus et al., 1995; Senechal and LeFevre, 2002), the age when regular storybook reading begins (DeBaryshe, 1993; Senechal and LeFevre, 2002), the word-knowledge children bring to the experience (Senechal et al., 1995; Hindman et al., 2008; Blewitt et al., 2009), the style of adult readers including the amount of elaboration on the meaning of new words (Ard and Beverly, 2004; Justice et al., 2005), and the complexity of adult readers' elaborations and questions about the book while reading (Whitehurst et al., 1988; Reese et al., 2003; Hindman et al., 2008; Blewitt et al., 2009). Typically, a more dialogic reading style by parents with open-ended questions, elaborations and repetitions which encourages the child to comment on the book (e.g., Whitehurst et al., 1988, 1994) can lead to better vocabulary learning. For instance, Ard and Beverly (2004) found that children learned more novel words from storybooks when they heard extra comments or questions about those words during the reading session. Hindman et al.'s (2008) longitudinal study of preschoolers reading with parents and teachers went one step further. They discovered that it was not just extra talk about the meanings of new words that affected word learning, but specifically talk that prompted children to recall, predict, and make inferences when reading stories that predicted gains in children's vocabulary. Thus, simply hearing new words in storybooks can help a child learn those words, but *actively* engaging with the words seems to provide an extra memory and learning boost.

So while we have amassed research on the role adult readers play in promoting word learning from storybooks and what characteristics of children may make them more receptive, we know much less about how the third part of this interaction, the book itself, helps to encourage word learning. Do some books prompt more active engagement on the part of the child? Are there features of certain types of books that might encourage more questions, more predictions, or simply more memorable words? In particular, what difference does rhyme, a ubiquitous storybook feature, make in helping children grow their vocabulary?

Almost thirty years ago, Moerk (1985) speculated on the potential benefits of rhyme, which, like repetition, increases predictability, and may thereby “simplify the analysis of the input for the child” (p. 553) making it easier for children to remember words from a story. He had observed mothers capitalizing on the predictability of rhyme when reading with children in a “testing and feedback” phenomenon that involved deliberately producing incomplete utterances leaving off the last word of a rhyme so that children could easily supply it (Moerk, 1972), yet he did not measure the effects of this kind of interaction on how well children might retain those rhyming words in their vocabulary. In fact, despite the ubiquity of rhyme in the life of a preschooler and the intuitions of parents and teachers (e.g., Kenney, 2005), only a small amount of empirical research has looked at whether children's books written in verse affect what is learned from them.

Rhyme is so common in the modern child's experience of shared storybook reading that we sometimes overlook it, but in a recent local survey of 160 parents of 2- to 4-year-old children, parents reported that on average rhyming books make up 38% of their home libraries, and of their own favorite books to read to their children 48% were rhyming (Read, Unpublished raw data). In fact, 20 of the “Top 100 children's books of all-time” (2013) for children ages 0–5 are in verse. Thus, a child who is read to regularly could be hearing hundreds of hours of rhymed language before the end of preschool. Children don't just hear a lot of rhyme they also clearly enjoy it. In Hayes et al.'s (1982) experiments, which pitted rhymed versus unrhymed versions of stories against one another for the purpose of testing children's recall, regardless of how well children remembered them, they consistently *liked* the rhyming versions of the stories significantly more than the prose versions. This seems unsurprising as it fits anecdotally with the first-hand experience of parents, teachers, and children's book writers, but *what* is the advantage to rhyme over prose besides the delight in it?

Experience with rhymes, whether gleaned from storybooks or simply recited orally, does correlate positively with other measures of language development. In a meta-analysis of 12 studies with 5299 3- to 6-year-olds, Dunst et al. (2011) found that nursery rhyme measures (e.g., knowledge of, or experience with specific nursery rhymes) were positively associated with several phonological and pre-literacy measures. The link from rhymes to reading outcomes is assumed to proceed through heightening phonological sensitivity (e.g., Bryant et al., 1989; Hayes, 2011). But our own query is more specific—could rhymes help foster language development because they make the vocabulary within them more predictable and thus easier to remember?

Two researchers in the last 40 years who have directly compared what children remember from prose vs. rhyming stories have found different things: Hayes and colleagues found 4- to 6-year-olds were hampered by rhyme when asked to recall the specific events of a story (Hayes et al., 1982; Johnson and Hayes, 1987; Hayes, 1999). However, Sheingold and Foundas (1978) found that 6-year-olds recalled just as many details of a story when they heard rhyming vs. prose versions, but that the rhymed stories gave children an advantage in memory for the sequence of events. In addition, children recalled more of the specific rhyming words of stories compared to other details (Hayes et al., 1982) and demonstrated better verbatim recall (Johnson and Hayes, 1987) despite decrements in overall paraphrase abilities.

Recently our own work has investigated the effects of rhymed versus unrhymed stories on 2- to 4-year-old children's ability to recall familiar animal names from a parent–child storybook reading session (Read and Macauley, submitted). We found not only that children remembered more target words when they had been presented in a rhymed story, but also that parents reading rhymed versions of the story would pause longer before naming a target animal and that in concert children would spontaneously guess the name of the animal during such pauses with significantly higher accuracy in the rhyming condition. Just as Moerk (1972) observed, a parent in this study would read *My floppy ears might look quite funny/if I were a hopping…* allowing the child time to supply *bunny!*, and then at the end of the book *bunny* was more easily recalled in this rhyme version than in a unrhymed version about the very same rabbit. This highlighted the way that rhyme supports predictability, but also how that predictability might be making the words themselves more memorable. Thus, rhyme, as well as being potentially engaging and playful, may be especially facilitative for recall because of how it can support active predictions about upcoming words.

Thus if rhyme is helpful to children for remembering such predictable familiar words, the question that follows is whether it could also aid in learning *new* words. This may be the case because stories written in verse increase the amount of overall predictability in the phrases children hear. Rhyme builds up an expectation for the sounds of upcoming words even if they are unfamiliar, and added to other cues like the story narrative or illustrations, can give a child clues about the form of a novel word at the end of a line even before it is read.

Recent research demonstrates how predictability gives children an edge in language comprehension and language learning. When words are predictable, for example when they are placed in frequent frames (Fernald and Hurtado, 2006) or common phrases like *brush your… teeth* (Arnon and Clark, 2011) or contextually specific phrases like *the pirate buried the… treasure* (Borovsky et al., 2012) then those words can be anticipated and identified more rapidly (Fernald and Hurtado, 2006; Borovsky et al., 2012) and produced correctly more often (Arnon and Clark, 2011). Predictability also influences how well children learn novel words. Ramscar et al. (2010) demonstrated the importance for word learners (whether they are children, adults, or computer models) of sequencing information in a way that facilitates prediction. They found that when a novel label is *preceded by* the discriminating features of a novel object then that label can be learned more easily, meaning that the most supportive thing we can do to teach a new word is to name it *after* the features that predict it.

Could a similar kind of predictability, the kind that rhyme adds to language as a redundant cue to the relevant features and the sound of a new word, also make that new word more memorable, and thus more learnable? The present study was designed to look not just at whether rhyme would aid memory for words from storybooks, but whether the specific way in which the rhyme sets up those new words would make a difference to how well children could retain and learn them. If rhyme makes words more memorable simply because it makes a storybook more engaging, then it would not matter where a new word was placed within a rhyming stanza, that word should still receive a boost. If rhyme makes words more memorable simply by highlighting them phonologically through the repetition of sound, then only novel words that are rhymed elements themselves should benefit. However, if the *predictability* that the rhyme creates is what boosts memory and learning then where the novel word is placed within a stanza should matter—putting the new word in the most predictable place at the end of the stanza after drawing attention to the novel features that distinguish it, and setting up an expectation for what it should sound like should be most beneficial for remembering and learning that new word. Thus, in two experiments we attempted to teach young children the novel names of several unfamiliar monsters under each kind of rhyming condition—one in which the monster name, though embedded within a rhyme, was not a rhyming element itself; one in which the monster name was a rhyming element but it was the first one heard; and one condition in which the monster name was the last rhyming element in a four-line stanza meant to provide the maximal amount of predictability.

## Experiment 1

### Methods

#### Participants

Twenty-six 2- to 4-year-old children participated (M_age_ = 39 months, *SD* = 9.5 months). Eighteen participants were girls and eight were boys. All were learning English as their primary language without any reported language delays or disabilities. Children were recruited through an on-campus preschool in Santa Clara, California, and tended to be from homes in which parents were college-educated and of moderate to high income levels. Children were randomly assigned to each of three conditions resulting in equal distribution of ages and genders among groups. Table 1 illustrates the age and gender breakdown of each condition group. We conducted a one-way analysis of variance in order to test for any significant difference in ages among condition groups, and found none, F_(2, 23)_ = 0.518, *p* = 0.602. One additional child participated but was excluded from all analyses due to inattentiveness and failure to participate in answering the test questions.

**Table 1:** **Age and Gender breakdown of each Condition Group in Experiment 1**.

**Condition**	**Mean age (months)**	**95% CI for age (months)**	**Number of girls/boys**
Non-rhyme	36.4	[29, 43]	7/3
Non-predictive rhyme	39.3	[31, 48]	6/2
Predictive rhyme	40.8	[33, 49]	5/3

#### Materials

Three “laptop stories”—one for each condition—were created for Experiment 1. Each story was a six-page rhyming introduction to a series of novel friendly monsters. Each page in the story featured a new monster on a white background, and the text of one rhyming stanza describing him. The monster pictures were bold-color cartoon-style drawings of monsters thought to be “cute” and “friendly” by a norming sample of five preschool-aged children. Each monster was illustrated to have a prominent feature (e.g., extra-large shoes, a big fuzzy nose) that was described within the rhyming stanza that accompanied his picture. The monster names were all one syllable, and each started with one of three consonant clusters and ended in a common rime. This was done so that we could create novel names that would, nonetheless, rhyme with features that would be common words even for children as young as two. (See Appendix for the full text of all the rhymes and monster names used in each condition).

Each of the rhymes was recorded by a single female reader using GarageBand^©^ recording software in a soundproof recording room. The reader used a child-friendly, evenly paced tone, and all stanzas were approximately the same volume and duration (between 11 and 12 s), as were all target monster names (between 700 and 950 ms).

The pages of each story were adapted into Powerpoint^©^ software as individual slides with each monster picture and recorded text coupled. After the story slides, a slide with just the text “Time to help out!” was added, followed by six more slides each with a pair of monsters from which a child would be asked to choose a target (never pairing two monsters with the same initial consonant cluster, and never presenting the same pair twice). Then, another plain text slide was inserted that read, “Can you say their names?” followed by the six monster pictures again presented one-by-one so that the child could be asked to produce a name for each. Animation was added to all the slides so that the “pages” would appear to turn like conventional print book pages from right to left automatically every 12 s.

#### Procedure

The study was conducted in a small quiet room meant to be inviting to children but relatively free of distractions at the children's preschool. Children whose parent had given consent for their participation were approached individually by the experimenter and asked if they would like to listen to a story “on the computer.” When children gave assent, they were shown to the testing room and seated at a small table with a 15” MacBook Pro® laptop. The video camera built into the laptop was turned on using the Photo Booth^©^ application so that the child's responses could be recorded. The stories were displayed using Powerpoint^©^ software that was set to automatically advance through the pages and play the audio recording of the text on each page, similar to an e-book. Each child was told that after the story the experimenter would ask some questions about the names of the monsters.

Children were randomly assigned to one of three between-subject conditions and heard the corresponding story. The pictures of the monsters and the order in which they were presented were identical in each condition; what differed among conditions was the placement of the monster name within the stanza and whether it was rhymed with the distinguishing features of the monster. In the non-rhyme condition, the monster name came within the first line and did not rhyme with the feature that distinguished it (though the stanza, itself, still rhymed). In the non-predictive rhyme condition, the name of the monster was a rhyming element, but came at the end of the first line, *before* the description of his unique rhyming feature. In the predictive rhyme condition each monster's name rhymed with his unique feature and came at the *end* of the four-line stanza.

After hearing the story, children were presented with monster pairs and asked to choose the named monster by pointing to his picture, e.g., “Which monster was the smooze?” as a test of novel name retention and identification. Then, children were shown pictures of each monster and asked the production test question, “What was this monster called?” For all questions, if children said that they didn't know they were asked if they wanted to make a guess, and regardless of how children answered, they were always praised for effort and reminded that this task was hard for the experimenter, which is why she needed their help.

Children's responses to both sets of questions were recorded during testing by one experimenter and then checked against the video-recordings later the same day by a second experimenter. Agreement between the two experimenters was greater than 98%. Children were given credit for a correct response on identification questions if they pointed first to the target monster or if they verbally described it, e.g., “He's the green one.” Children were given credit for a correct response on production questions if they said the name of the monster or a close approximation that rhymed, e.g., “He's a smooze” or “a mooze.” For each child we tallied the number of correct responses (out of six) for both identification and production test questions separately.

### Results

Descriptive statistics for children's age and performance on the task in each condition are given in Table 2.

**Table 2:** **Response performance by Condition in Experiment 1**.

	**Condition**		
	**Non-rhyme rhyme mean 95% CI**	**Non-predictive rhyme mean 95% CI**	**Predictive rhyme mean 95% CI**
% Correct monster identifications	40_a_ [27, 53]	58_ab_ [45, 72]	65_b_ [45, 83]
% Correct monster name productions	0	5 [−3, 12]	8 [0, 17]

CI, confidence interval; means that do not share subscripts differ from one another at p < 0.05.

In order to investigate the effects of rhyme and rhyme placement on children's success in our tasks we did separate analyses of covariance (ANCOVAs) for the identification task and for the production task. For the identification task, an ANCOVA with condition as a between subjects' factor and age as a possible covariate revealed a significant effect of condition on performance in the identification task, *F*_(2, 22)_ = 4.781, *p* = 0.02 but no significant effect of age, *F*_(1, 22)_ = 1.912, *p* = 0.18, meaning that children's ability to correctly identify a monster in our task was affected by which condition they were in regardless of their age. The size of the effect of condition on identification scores was moderate, η^2^ = 0.26. *Post-hoc* Tukey HSD tests were conducted to test children's performance in each condition pair-wise, and these revealed that the overall effects for the identification test comparison were driven by significantly greater performance in the predictive rhyme condition than the control non-rhyme condition, *p* < 0.05 with performance in the non-predictive rhyme condition falling in between the other two conditions without differing significantly from either.

For the production task, an ANCOVA with condition as a between subjects' factor and age as a possible covariate revealed no significant effect of condition on performance in the production task, *F*_(2, 22)_ = 2.164, *p* = 0.14 and only a marginal but not significant effect of age, *F*_(1, 22)_ = 2.960, *p* = 0.10, meaning that older children were not significantly more likely to successfully name a monster in any condition over another, and that while age may have had some effect it was also not a significant predictor of children's ability to produce monster names correctly. In addition, production test scores should be interpreted with caution, as there was clearly a “floor effect” skewing the distribution given that 20 out of the 26 participants could not name one monster.

### Discussion of experiment 1

These results indicate that the condition that promoted the best retention of the novel monster names was, as we hypothesized, the condition that provided children with the maximal amount of predictability. In other words, when the monster name came after the feature that distinguished that monster and when the sound of the monster's name could be predicted from three prior rhyming elements, then the name-monster mapping was easiest for children to recall. The finding that performance of children in the non-predictive rhyme condition did not differ from either of the other conditions hinted that simply using the novel name as a rhyming element at the beginning of the stanza, where it also received line-final stress but was less predictable may have not have been enough to make it more memorable than a non-rhyming novel name. However, because the difference between performance in the predictive and non-predictive rhyme conditions was not statistically significant, we could not be certain that placement alone (rather than a combination of placement and rhyme) was the important contributing factor. In order to focus in on the comparison between the most predictable rhymed novel words and the less predictable but still rhymed novel words, and to reduce the amount of extraneous variability inherent in a between subjects comparison of young children, we designed Experiment 2 as a within-subjects comparison of just the predictive and non-predictive rhyme conditions.

In addition, perhaps unsurprisingly, identifying correct monsters when given their names in a two-alternative forced choice test appeared to be easier for children than producing their names spontaneously. The production test may have been especially taxing for young children because of their language or memory skills in general but also because young children may not have been comfortable answering questions and speaking out loud to an unfamiliar adult. Producing the monster names required more memory of the monsters but also more verbal ability and more confidence than simply identifying the monsters when named. To improve the naturalness of the experience for children and potentially their comfort level we also moved to a parent–child reading of the books in Experiment 2 rather than a pre-recorded narration of the book.

We believed bringing parents into the lab along with their children would emulate the child's more typical storybook reading experience, and would allow children and their parents to control the pacing in a more natural way that might help them remember and learn the monster names more successfully. Having the parents read the stories to their children also enabled us to consider the impact of reading style variables (e.g., emphasis on new vocabulary, amount of extra-textual talk) on how well the monster names were retained from this type of storybook.

So, in Experiment 2, we attempted to improve on Experiment 1 by investigating performance in a new group of children of the same age who heard the same predictive and non-predictive rhymes from Experiment 1, but in a within-subjects design. And, instead of hearing the rhymes pre-recorded, children's caregivers read the stories naturally allowing for variation in overall timing, pauses, emphasis and extra-textual talk as they might use in a more common and comfortable storybook experience at home.

## Experiment 2

### Methods

#### Participants

Twenty-eight 2- to 4-year-old children participated (M_age_ = 40 months, *SD* = 8 months). Sixteen of the participants were girls, 12 were boys. All were learning English as their primary language without any reported language delays or disabilities. Each participant brought one parent with them (three fathers, the rest mothers) who had been recruited through parenting groups and an on-campus preschool in Santa Clara, California. Participants tended to be from homes in which parents were college-educated and of moderate to high income levels. All children in the study were reportedly read to daily by the parent who accompanied them. Two additional children participated but were not included in any analysis – one child's video recording failed because of technical error, and one child was excessively distracted during the story and could not complete testing.

#### Materials

Four “laptop stories” were created for Experiment 2 using the pictures and rhymes from Experiment 1 with the addition of two new monsters and their accompanying rhymes (see Appendix). Each story in Experiment 2 included four monsters presented with the text from the predictive rhyme condition and four monsters presented with the text from the non-predictive rhyme condition interspersed in one of four orders—order A1 was the reverse of order A2 (e.g., if the groze was the first monster presented in A1, it was the last monster of A2), and orders B1 and B2 were the condition inverses of A1 and A2 (e.g., if the groze was presented in a predictive rhyme in A1, it was presented in a non-predictive rhyme in B1). As in Experiment 1, stories were created and presented using Powerpoint^©^ software. Test pages for the identification and production tests were the same as in Experiment 1 with the two new monsters added. In Experiment 2 there was no recorded narration and there was no automatic timing added, as parents were asked to read the stories to their children at their own pace.

#### Procedure

The study was conducted in a small quiet room meant to be inviting to children but relatively free of distractions in an academic building on campus. At the beginning of the session there was a short play period, where children were invited to color a picture or play with a puzzle while the procedure and consent form were explained to the parent. Afterward, each child was asked if he or she would like to listen to his or her mom or dad read a story “on the computer.” Children and their parents sat at a small table with a 15” MacBook Pro® laptop. As in Experiment 1, the video camera built into the laptop was turned on so that the story reading and child's responses could be recorded, and the stories were displayed using Powerpoint^©^ software, so that the parents could easily read page-by-page at their own pace as they would with a conventional print storybook. Each parent was told to read the story naturally, the way they would at home, and each child was told that afterwards the experimenter would ask some questions about the animals in the story. Children were randomly assigned to one of the four orders and the corresponding story was opened for their parent to read.

Once the parent had read the story, the experimenter then asked the child if he or she liked the story and could help her remember some of the monster names. With the child's assent, the experimenter proceeded with the test questions by asking the child the two-choice identification questions and then the open-ended production questions in the same manner as in Experiment 1. Parents were seated behind children during this testing period so as not to give any cues (intentional or unintentional) about the monsters in question.

#### Measures

We were primarily interested in the effect of condition on how well children retained the novel monster names that they heard in the story, and so as in Experiment 1, we calculated identification and production scores for each child based on how many monsters they correctly chose or named during testing. However, we also took measures of each parent's reading in order to capture some of the individual variations that each parent and child brought to the storybook reading experience and to investigate those variables' impact on retention.

For each parent, we took two measures using just the audio of the story reading session converted into Audacity® sound editing software files for precision. First, we measured to the hundredth of a second the duration of the pause that preceded naming each target monster (e.g., the length of time from the offset of “called a” to the onset of “smooze”). Pause duration was averaged for each parent across the four monsters within each condition. This measure was meant to investigate whether there might be a difference between conditions in the amount of time parents allowed a child to prepare for and anticipate an upcoming monster name and/or whether any such pause was correlated with later retention (e.g., Read and Macauley, submitted). Second, we measured the duration of each novel target label (e.g., the time it took the parent to say “smooze”). While in both conditions, the monster names were expected to receive sentence-final stress and perhaps emphasis because of their novelty (e.g., Fernald and Mazzie, 1991; Clark, 2010), this measure was meant to investigate whether the emphasis parents placed on the name of the animals differed based on whether they were in the predictive rhyme or non-predictive rhyme condition and/or was correlated with later retention. And third, we measured the average overall duration of the rhyme stanza for each parent in each condition as a way of investigating whether there was an effect of condition on parents' pacing and/or whether it correlated with later retention.

Lastly, we also measured parents' extra-textual talk in Experiment 2 by counting the total number of utterances that each parent made between starting the first stanza and the beginning of the test questions. Extra-textual talk included comments such as, “Oh he's cute,” “wh” questions such as, “What is that guy doing?” and responses to children's questions, but not self-corrections or comments directed to the experimenter. This measure was meant to capture the total amount of story-related commentary as an indicator of the parents' reading style (e.g., Reese et al., 2003) in order for us to investigate whether reading style differed by condition and/or correlated with later retention.

Each of these measures was double-coded by trained research assistants to establish inter-rater reliability greater than 90%. Whenever there was disagreement, a discussion among the raters and the primary investigator settled the final measure.

### Results

Descriptive statistics for children's performance on the task and measures of parents' reading in each condition are given in Table 3.

**Table 3:** **Parents' reading measures and children's performance by Condition in Experiment 2**.

	**Condition**	
	**Non-predictive rhyme mean 95% CI**	**Predictive rhyme mean 95% CI**
**PARENT MEASURES**		
Pause duration (ms)	95 [27, 163]	249^*^ [136, 361]
Target duration (ms)	754 [697, 812]	817 [759, 875]
Stanza duration (s)	12.7 [11.2, 14.2]	11.8 [11.1, 12.6]
Extra-textual utterances	4.0 [1.5, 6.5]	3.9 [1.2, 6.5]
**CHILD MEASURES**		
% Correct monster identifications	58 [49, 67]	72^*^ [65, 80]
% Correct monster name productions	7 [3, 12]	11 [5, 16]

CI, confidence interval.

* p < 0.01 for difference between means of each condition.

The main question of Experiment 2 was whether children differed in their ability to correctly identify the novel monsters between conditions. An ANCOVA with condition as a within subjects' factor and age as a possible covariate revealed a significant effect of condition on performance in the comprehension task, *F*_(1, 26)_ = 9.258, *p* = 0.005, and no comprehension by age interaction, *F*_(1, 26)_ = 0.002, *p* = 0.96. Thus, children identified significantly more monsters correctly in the predictive rhyme condition than in the non-predictive rhyme condition, and the effect between conditions was moderately large, cohen's *d* = 0.66. Children identified more monsters correctly when they had been presented at the end of the stanza than when they were introduced at the beginning.

There was not, however, a significant difference between conditions in monster name productions. An ANCOVA with condition as a within subjects' factor and age as a possible covariate revealed no significant effect of condition on performance in the production task, *F*_(1, 26)_ = 0.262, *p* = 0.61, and no production by age interaction, *F*_(1, 26)_ = 0.000, *p* = 0.99. As in Experiment 1, there seemed to be a “floor effect” skewing the distribution of production scores such that 13 of the 28 children (almost half) never produced a single name.

Within-subjects comparisons of the parent reading measures in Experiment 2 uncovered no differences between the predictive and non-predictive rhyme conditions (all *p*'s > 0.06), except for in the duration of the pause parents took before they named the monster. Parents paused before naming a monster significantly longer when reading predictive rhymes compared to non-predictive rhymes, *t*_(27)_ = 2.81, *p* < 0.01 (two-tailed) and the size of this effect was moderate, cohen's *d* = 0.66. In fact, as can be seen in Table 2, parents' pause durations in the predictive rhyme condition were more than two-and-a-half times longer than in the non-predictive rhyme condition, even though in both conditions the monster names themselves were not different in duration or emphasis, and despite no difference in the overall pacing of the stanza between conditions.

Finally, when we investigated whether parents' reading variables had any direct relationship with children's monster name retention, we found no significant correlations between any measures of parents' reading and the numbers of monsters correctly identified or produced by children. However, even though there was not a significant correlation between pause duration and correct monster identification overall (*r* = 0.24, *p* = 0.22, two-tailed) we wanted to look more closely at the subgroup of children who had heard pauses longer than 250 ms on average. Qualitatively, a pause shorter than 250 ms was imperceptible without sound-editing software. When we split children into two groups based on whether their parents' average pause length was greater than or less than 250 ms, there were 10 children who heard audible pauses before the monster names (all in the predictive rhyme condition), and notably, those 10 children showed a significant predictive rhyme advantage—this subgroup who had heard pauses greater than 250 ms had a mean difference score for correct identifications in the predictive rhyme condition minus the non-predictive rhyme condition of 0.80, significantly greater than 0, *t*_(9)_ = 2.45, *p* < 0.05; whereas those 18 children who did not hear an audible pause had an average difference score of 0.22, not different from 0, *t*_(17)_ = 0.747, *p* = 0.47. This meant that when children actually heard a pause before the novel monster name they remembered almost one extra monster from the predictive rhyme condition compared to the non-predictive rhyme condition.

#### Discussion of experiment 2

With Experiment 2, we were able to see that it does indeed matter whether a novel word comes at the beginning or the end of a rhyming stanza for how well it can be remembered and learned. The main finding demonstrated that even when the same parents and children were reading a storybook, when the monster names came at the end of the stanza they were more memorable than when they came at the beginning. Monster names in both conditions received the support of rhyme, and even unique rhymed features that differentiated the monster, and monster names in both conditions received line-final prosodic emphases not differing in their durations. It appeared that *location* of the monster name within the stanza, on top of rhyme, distinguishing features and emphasis influenced differences in children's retention. Further, for those children whose parents paused just a quarter of a second or longer before reading the monster name in the predictive rhyme condition, there was an extra memory boost for those predictable monsters. We interpret this as a possible link between rhyme, predictability, and retention. For children who heard that little pause before the novel monster's name, there may have been an additive effect of time plus predictability, giving them an extra moment to anticipate the upcoming monster's name and an edge in remembering it a few minutes later.

## General discussion

This study gave us a new view on the effect of rhyme on how well children can remember and learn novel words from stories. In Experiment 1 the differences found in monster name identification between the non-rhyme and the predictive rhyme conditions supported the few previous findings that rhyming words can make them more memorable for children (e.g., Hayes et al., 1982; Read and Macauley, submitted) showing that this is also the case for novel words that are rhymed with the features that help to distinguish them. Experiment 2 added to this finding by demonstrating that not only rhyming the novel words, but also placing them at the end of a stanza after the build-up of some anticipation was especially helpful.

Experiment 2 was also meant to be more natural for children, since their own parents read the stories with them. While the tasks were not directly comparable across the two experiments, we believe that children in Experiment 2 had more support with their parents present and reading to them, and that this may have encouraged a more comfortable storybook reading environment. For the purposes of this exploratory work, there was value-added in allowing parents to read the stories themselves free of constraints, in order for us to observe what techniques of reading parents spontaneously used with these rhymes. After all, it is that three-way interaction among parent, child, *and* book that enables word learning to occur. Of course now having seen how parents make use of strategic pauses in just the places we hypothesize may be helpful to young listeners, the next step of future research would be to empirically test the effects of deliberate pausing (or lack thereof) in a more controlled reading, such as that used in Experiment 1.

So, why does putting the monster name at the end of a stanza really make a difference? In our view, it is not simply extra emphasis on new words at the end of the stanza since line-final *and* stanza-final words were emphasized and elongated, and not simply a recency effect since testing occurred after many monster names had been heard at the beginning or end of stanzas. Our hypothesis is that it is the build-up to those novel words, their extra predictability, which encourages more engagement with them on the part of the child. A child may not be able to predict the exact name of a new monster (or any novel word for that matter) upon the first reading of a story, but when the new name comes at the end of the stanza the child might better be able to anticipate that *something* is coming that will *sound like* the previous line-endings. That anticipation may encourage attention, even some active prediction, and certainly may make the new name “stickier” as it is heard. The novel word at the end of the stanza just fits, like the last piece put into a puzzle, and is therefore boosted by how much the child has anticipated it. It is important to note here that because we think the *anticipation* of the rhyme is what may increase attention and make the novel stanza-final words memorable, this begs the interesting empirical question of whether even if the rhyme scheme were broken and the monster name failed the rhyme, whether the child would still find it memorable (or even *more* memorable?). Certainly, learning can occur when what we predict turns out to be wrong (e.g., Ramscar et al., 2010).

In the current study we have begun an initial exploration of three factors—rhyme, placement, and pausing—on predictability and the impact predictability has on novel word retention. We have not, however, begun teasing each of these factors apart, but rather have compared conditions that afforded little predictability (the non-rhyme condition of Experiment 1) to those that accumulated predictability. Because of this we cannot necessarily compare the individual factors to one another or yet understand how they might be interacting. In the monster rhymes of this study, we gave children as many advantages as we could to support the challenging task of mapping so many novel items in such a short story. In addition to the sound of the monster names being predictable from the preceding rhymes, we also built in unique features of each monster that could help distinguish him and highlighted those with rhyme. This, in accord with the feature-label ordering found to be most helpful by Ramscar et al. (2010), added a second layer of predictability to the stanza-final monsters in the predictive rhyme conditions. In future research, it will be valuable to tease apart these sources of predictability to assess each one's unique contribution. However, we were encouraged by these initial results demonstrating that added predictability in general could benefit children's retention.

In any study of word retention we must also consider the different challenges of hearing a new word and being able to pick out a referent that goes with it (i.e., receptive vocabulary acquisition) and the often more difficult task of encountering a referent and being able to produce the new word (i.e., expressive vocabulary acquisition). In both Experiments 1 and 2, children found it very difficult to correctly produce the monster names themselves, averaging less than a single monster in any condition. Thus, their “learning” of the monster names as demonstrated by the identification task was certainly only a quick mapping—just the beginning stage of really knowing any new word. The challenge children faced in the production task was not entirely surprising, as we already know that there are differences in how well children gain vocabulary receptively versus productively from storybooks (e.g., Senechal, 1997), and in the real-world of shared storybook reading parents usually repeat books (and the novel words within) many times before children add those words to their productive vocabularies (e.g., Snow and Goldfield, 1983; Horst, 2013). Thus, it would be a key next step to investigate how the advantage for predictably placed, rhymed, stanza-final, novel words would play out after multiple readings in which the child would not only begin to find the novel words more familiar but also increasingly predictable.

Additionally, what children, themselves, bring to the task of word learning from storybooks is also important to consider. While the age range of participants was wide in the two experiments presented here, age was not, in itself, a significant factor. Age may have played a marginal role in children's successes in the production task, but because scores were so low in that task—basically at floor, we would caution such an interpretation. Often age in such a wide span of children is a proxy for language ability, though since we were not able to test children's overall vocabulary comprehension or production abilities with standardized tests here, we cannot know whether the effects of rhyme, and rhyme placement might have differed for children with high versus low (relative to their age) language skill. However, this also would be an important next step for future research. Ideally, a more diverse sample with a wider range of language ability and familiarity with shared book reading in future research would allow us to assess how these factors influence children's ability to take advantage of the predictability of rhyme.

Finally, this work on rhyme may beg the question that some of our parent participants have asked—should all children's books rhyme? Or even, should all rhyming books place the novel vocabulary at the end of a stanza? The answer is of course, no—some books just don't lend themselves to rhyme, and there is much more to be learned from shared storybook reading besides new vocabulary. But, if the singular goal of a book or of a parent or teacher is to teach a few specific words it certainly wouldn't hurt. In the well-known Dr. Seuss classic *Did I ever tell you how lucky you are?* (Seuss, 1973) the following stanza describes a particularly unlucky young man:

Suppose, just suppose, you were poor Herbie Hart,

who has taken his Throm-dim-bu-lator apart!

He *never* will get it together, I'm sure.

He never will know if the Gick or the Goor

fits into the Skrux or the Snux or the Snoor (p. 13).

Our findings in this study cannot speak to whether a child will sympathize with Herbie or find this funny, but they do predict that of all these nonsense words, *Snoor* should be best remembered, and *if* it were a real piece of equipment might then become the easiest to learn.

## Author note

This research was not financially supported, but I am indebted to the many children and parents who freely volunteered and participated in this study and the partnership of Kids on Campus Preschool at Santa Clara University. I would also like to acknowledge the help and feedback received from Dr. Lisa Whitfield, and research assistants Jennifer Coleman, Megan Macauley, Briana Mitchell, and Andrew Weaver.

### Conflict of interest statement

The author declares that the research was conducted in the absence of any commercial or financial relationships that could be construed as a potential conflict of interest.

## Appendix

### Text of rhymes used in each condition of experiment 1 (target words in bold)

#### Non-rhymes

Here's a **smooze** who likes to cook and on his head is a useful hook where he can keep his recipe book so when he's hungry he just takes a look

Here's a **groze** you cannot lose wherever he goes he leaves some clues because he wears two huge red shoes his footprints are always real big news

This smart **flar** always knows when he's near a skunk or near a rose because he has a giant nose he sniffs when he comes and sniffs when he goes.

This **greers** can go very far when he rides in his super car he's faster than a shooting star he's so quick he's hard to draw

This sweet **smai** has no fears because he has perfect heart-shaped ears and the nicest sounds are all he hears this lovely monster always cheers

When this **flook** looks at the sky he uses his one big bright eye to see all the stars way up so high and the airplanes going by

#### Non-predictive rhymes

This clever monster's called a **flook** He really likes to bake and cook and on his head is a useful hook to help him find recipes in his book

This funny monster's called a **smooze** He's someone you cannot lose because he wears two huge red shoes wherever he goes he leaves some clues

This sniffing monster's called a **groze** And this guy, he always knows when he's near a skunk or near a rose because he has a giant nose

This speedy moster's called a **flar** He can go so very far when he rides in his super car he's faster than a shooting star

This lovely monster's called a **greers** He's super sweet and has no fears because he has perfect heart-shaped ears and the nicest sounds are all he hears

This dreamy monster's called a **smai** When he looks up at the sky He uses his one big bright eye To see all the stars way up so high

#### Predictive rhymes

Here's a monster who likes to cook and on his head is a useful hook to help him find recipes in his book this clever monster's called a **flook**

Here's a monster you cannot lose because he wears two huge red shoes wherever he goes he leaves some clues this funny monster's called a **smooze**

This smart monster always knows when he's near a skunk or near a rose because he has a giant nose this sniffing monster's called a **groze**

This monster can go very far when he rides in his super car he's faster than a shooting star this speedy moster's called a **flar**

This sweet monster has no fears because he has perfect heart-shaped ears and the nicest sounds are all he hears this lovely monster's called a **greers**

When this monster looks at the sky He uses his one big bright eye To see all the stars way up so high This dreamy monster's called a smai

### Additional rhymes used in experiment 2

#### Non-predictive rhymes

This soaring monster's called a **trings** Up in the air he smiles and sings He flies so high with his famous wings Maybe he can teach the birds some things

This daring monster's called a **traul** This guy will not ever fall He's balanced on his shiny ball Up there he's feeling pretty tall

#### Predictive rhymes

Here's a monster who smiles and sings And flies so high with his famous wings Maybe he can teach the birds some things This soaring monster's called a **trings**

This monster will not ever fall He's balanced on his shiny ball Up there he's feeling pretty tall This daring monster's called a **traul**
